# *Salmonella* Enteritidis Subunit Vaccine Candidate Based on SseB Protein Co-Delivered with Simvastatin as Adjuvant

**DOI:** 10.3390/pathogens11040443

**Published:** 2022-04-07

**Authors:** Xilong Kang, Tingting Huang, Huanhuan Shen, Chuang Meng, Xinan Jiao, Zhiming Pan

**Affiliations:** 1 Jiangsu Key Laboratory of Zoonosis, Yangzhou University, Yangzhou 225009, China; xlkang@yzu.edu.cn (X.K.); tingtingh309@163.com (T.H.); huanhshen@163.com (H.S.); mengchuang@yzu.edu.cn (C.M.); 2 Jiangsu Co-Innovation Center for the Prevention and Control of Important Animal Infectious Diseases and Zoonoses, Yangzhou University, Yangzhou 225009, China; 3 Key Laboratory of Prevention and Control of Biological Hazard Factors (Animal Origin) for Agrifood Safety and Quality, MOA, Yangzhou University, Yangzhou 225009, China; 4 Joint International Research Laboratory of Agriculture and Agri-Product Safety of the Ministry of Education, Yangzhou University, Yangzhou 225009, China

**Keywords:** *S*. Enteritidis, subunit vaccine, SseB, simvastatin, immune response, immune protection

## Abstract

*Salmonella enterica* serovar Enteritidis (*S*. Enteritidis) is an important zoonotic pathogen that can lead to diarrhea and systemic infections in humans and mortality in animals. This is a major public health issue worldwide. Safe and effective vaccines are urgently needed to control and prevent *Salmonella* infection. Subunit vaccines are safe and provide targeted protection against *Salmonella* spp. Here, we developed and evaluated an *S*. Enteritidis subunit vaccine candidate, the rHis-SseB adjuvant with simvastatin. We amplified the *SseB* gene from *S*. Enteritidis C50041 genomic DNA and expressed the recombinant proteins rHis-SseB and rGST-SseB using the *Escherichia coli* system. Western blotting confirmed the immunoreactivity of recombinant proteins rHis-SseB and rGST-SseB with antisera against *Salmonella* Enteritidis C50041. In a mouse model of intramuscular vaccination, co-immunization with rHis-SseB and simvastatin significantly enhanced both the SseB-specific antibody titer in serum (humoral immune response) and splenic lymphocyte proliferation (cellular immune response). Co-immunization with rHis-SseB and simvastatin provided 60% protection against subsequent challenge with the *S*. Enteritidis C50041 strain and decreased bacterial colonization in the liver and spleen. These findings provide a basis for the development of an *S*. Enteritidis subunit vaccine.

## 1. Introduction

*Salmonella* is an intracellular pathogen of gram-negative bacteria that consists of more than 2600 serotypes [1,2]. *Salmonella* infection can cause salmonellosis, a severe foodborne disease in humans and animals [3]. Salmonellosis is considered the most important zoonotic and foodborne illness worldwide [4]. It has been reported to cause approximately 70–80% of foodborne disease outbreaks in China [5]. In recent years, *Salmonella enterica* serovar Enteritidis (*S*. Enteritidis) has become one of the most common types of salmonellosis in humans and animals [6]. Humans infected with *S*. Enteritidis have diarrhea and systemic infections [7,8]. Infection of young chicks with *S*. Enteritidis can cause a high mortality rate and lead to heavy economic losses [9]. *S*. Enteritidis infections are still frequent despite the number of control and prevention measures performed [10], and new control and prevention methods are urgently needed.

Vaccination is an important and effective way to control and prevent salmonellosis [11,12]. There are three types of *Salmonella* vaccines: inactivated vaccines, live-attenuated vaccines, and subunit vaccines. Various live-attenuated and killed *S*. Enteritidis vaccines are used commercially worldwide and have been shown to be effective. For example, Layermune SE^®^ [13] and TAD *Salmonella* vac^®^ E [14] are inactivated and live-attenuated vaccines, respectively, that control *S*. Enteritidis. However, the live-attenuated vaccine has disadvantages that may reverse its virulence or interfere with wild-type *Salmonella* testing [15]. The inactivated vaccine also has many disadvantages in that it may be quickly eliminated by the host and cannot induce cellular immune response [16]. It also poses a risk because of the possibility of incomplete inactivation of the killed vaccine. Unlike inactivated or live-attenuated vaccines, the subunit vaccines are composed of defined antigens and are safe and easy to operate [17]. The development of subunit vaccines for *Salmonella* is a new method of controlling salmonellosis.

The development of bacterial subunit vaccines is a hot research topic. Several *Salmonella* proteins have been identified as *Salmonella* subunit vaccine antigens [17,18,19,20,21]. Meenakshi et al. immunized chickens with the outer membrane proteins of *S*. Enteritidis, and bacterial shedding was significantly reduced [18]. In another study, Toyota-Hanatani et al. demonstrated that the load of *S*. Enteritidis was significantly decrease in the chicken cecal contents after immunization with FliC [19]. In addition, other proteins, such as type I fimbriae and SPI-1 and SPI-2 proteins, have been used as subunit vaccine candidate antigens [17,20,21]. These subunit vaccine candidate antigens have the same characteristics as those expressed on the bacterial cell surface or have very important virulence features [17]. The *Salmonella* SseB protein is a virulence protein encoded by *Salmonella* pathogenicity island 2. The SseB protein in *Salmonella* is localized on the bacterial surface [22]. Because of these characteristics, SseB may be used as a subunit vaccine. The immunogenicity of subunit vaccines also needs to be strengthened using appropriate adjuvants [23]. Simvastatin, a lipophilic statin, can inhibit mevalonate pathway resulting in arrested endosomal maturation, prolonged antigen retention, enhanced antigen presentation, and T cell activation [24]. It has been demonstrated that simvastatin has highly effective adjuvant activity [24]. Compared with alum, MF59, et al., simvastatin has higher adjuvant activity [24].

In this study, we cloned the *SseB* gene from *S*. Enteritidis C50041 and expressed the recombinant proteins rHis-SseB and rGST-SseB using the *Escherichia coli* system. We confirmed the immunoreactivity of rHis-SseB and rGST-SseB by western blotting using antisera against *Salmonella* Enteritidis C50041. Furthermore, we evaluated rHis-SseB mixed with simvastatin as a subunit vaccine by measuring antibody response, splenic lymphocyte proliferation, protective efficacy, and bacterial colonization in organs.

## 2. Results

### 2.1. Expression, Purification, and Identification of Recombinant Proteins rHis-SseB and rGST-SseB

**Figure 1:**
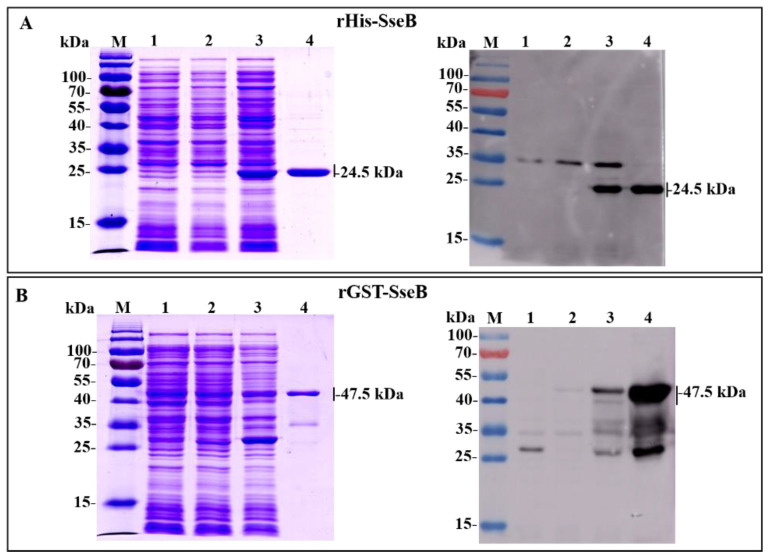
Expression and purification of recombinant proteins rHis-SseB and rGST-SseB in *E. coli* BL21(DE3) using SDS-PAGE and western blotting. (**A**) SDS-PAGE and western blotting analysis of the expression and purification of rHis-SseB. Western blotting was analyzed using a monoclonal anti-His tag. Lanes: M, protein molecular weight markers; 1, lysate supernatant of *E. coli* BL21 (DE3) harboring pCold empty vector induced with IPTG; 2, lysate supernatant of *E. coli* BL21 (DE3) harboring pCold-*SseB* induced without IPTG; 3, lysate supernatant of *E. coli* BL21 (DE3) harboring pCold-*SseB* induced with IPTG; 4, purified rHis-SseB protein. (**B**) SDS-PAGE and western blotting analysis of the expression and purification of rGST-SseB. Western blotting was analyzed using a monoclonal anti-GST tag. Lanes: M, protein molecular weight markers; 1, lysate supernatant of *E. coli* BL21 (DE3) harboring pGEX-6p-1 empty vector induced with IPTG; 2, lysate supernatant of *E. coli* BL21 (DE3) harboring pGEX-6p-1-*SseB* induced without IPTG; 3, lysate supernatant of *E. coli* BL21 (DE3) harboring pGEX-6p-1-*SseB* induced with IPTG; 4, purified rGST-SseB protein.

Expression of the recombinant proteins rHis-SseB and rGST-SseB in *E. coli* BL21(DE3) was induced by the addition of IPTG. SDS-PAGE results show that the expected bands, with molecular masses of 24.1 kDa (Figure 1A) and 47.5 kDa (Figure 1B), corresponding to rHis-SseB and rGST-SseB, respectively, were observed in lysate supernatant of bacteria. rHis-SseB and rGST-SseB were purified from 500-mL of cell culture using a Ni-NTA affinity column. Purified rHis-SseB (Figure 1A) and rGST-SseB (Figure 1B) were confirmed by SDS-PAGE and western blotting with anti-His or GST tag antibodies.

### 2.2. Immunoreactivity of Recombinant Proteins rHis-SseB and rGST-SseB

**Figure 2:**
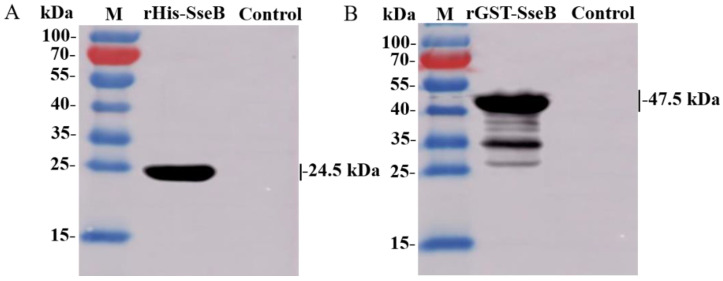
Immunoreactivity of recombinant proteins rHis-SseB and rGST-SseB. rHis-SseB (**A**) and rGST-SseB (**B**) proteins were analyzed by western blotting using antisera against *Salmonella* Enteritidis C50041.

To analyze the immunoreactivity of rHis-SseB and rGST-SseB proteins, western blotting was performed using antisera against *Salmonella* Enteritidis C50041 as the primary antibody. The results show that the rHis-SseB and rGST-SseB proteins reacted with antibodies against *Salmonella* Enteritidis. The band was observed at the 24.1 kDa region corresponding to the rHis-SseB molecular masses (Figure 2A) and the 47.5 kDa region corresponding to the rGST-SseB molecular masses (Figure 2B).

### 2.3. Simvastatin-Enhanced Antibodies Induced by rHis-SseB in Serum

**Figure 3:**
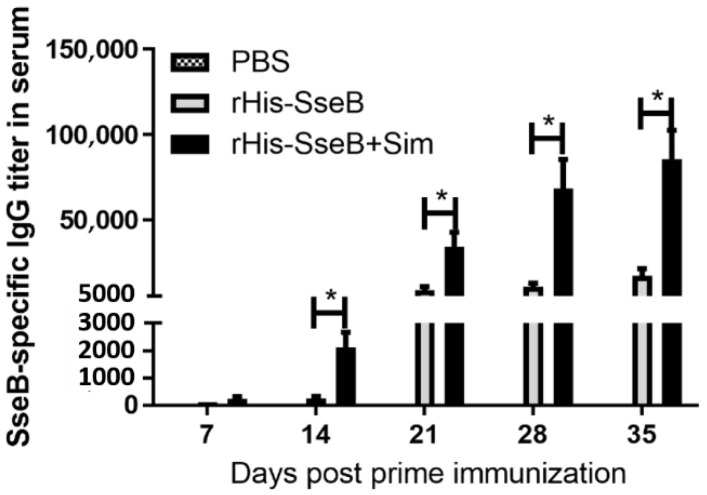
SseB-specific IgG titers in serum using indirect ELISA. BALB/C mice were immunized either with rHis-SseB, rHis-SseB mixed with simvastatin, or PBS twice on days 0 and 14. Serum was collected on days 7, 14, 21, 28, and 35 for analysis of SseB-specific IgG titers by ELISA. rGST-SseB was used as coating antigen when ELISA was performed. Data are presented as mean ± SEM, * *p*  < 0.05.

To evaluate the antibody response induced by rHis-SseB, mice were immunized twice with rHis-SseB, rHis-SseB mixed with simvastatin, or PBS. Following immunization, an indirect ELISA was used to measure SseB-specific IgG titers in the serum. The results show that immunization with rHis-SseB mixed with simvastatin induced significantly higher SseB-specific IgG titers (*p* < 0.05) than immunization with rHis-SseB alone or PBS on days 14, 21, 28, and 35 (Figure 3). The SseB-specific IgG antibody in the rHis-SseB+Sim group was first detected (mean titer 233) on day 7 post-prime injection, whereas the SseB antibody in the rHis-SseB group was first measured (mean titer 266) on day 14 (Figure 3). Higher SseB-specific IgG titers were quickly induced after boost immunization in both the rHis-SseB+Sim and rHis-SseB groups (Figure 3). The titer in the rHis-SseB+Sim group was elevated almost 40-fold after the second immunization compared to that after the first immunization.

### 2.4. IgG Subtype Induced by rHis-SseB in Serum

**Figure 4:**
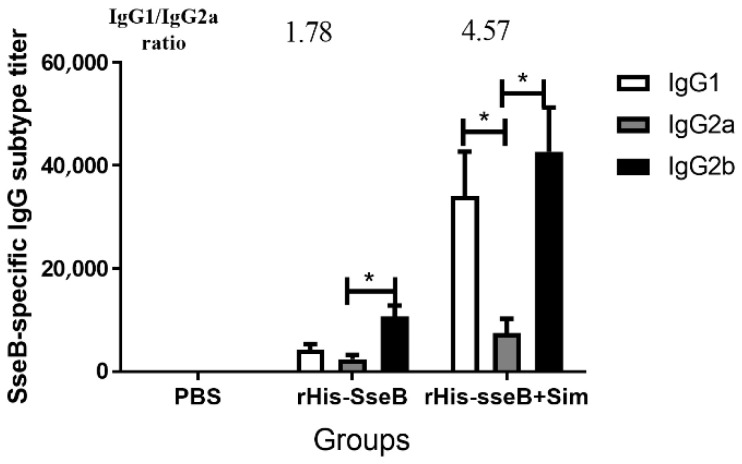
SseB-specific IgG subtype in serum 14 days after the second immunization. SseB-specific IgG1, IgG2a, and IgG2b titers were measured by indirect ELISA. rGST-SseB was used as coating antigen when ELISA was performed. Data are presented as mean ± SEM, * *p*  < 0.05.

We investigated the IgG subtypes, including IgG1, IgG2a, and IgG2b, 14 days after the second immunization, and calculated the IgG1/IgG2a ratio. In the rHis-SseB+Sim group, the SseB-specific IgG1 titers were prominently above the IgG2a titers (*p* < 0.05), and the IgG1/IgG2a ratio was 4.57 (Figure 4). The SseB-specific IgG2b titers were also prominently above the IgG2a titers (*p* < 0.05) in the rHis-SseB+Sim group (Figure 4). Mice immunized with rHis-SseB alone induced SseB-specific IgG2b titers higher than IgG2a (*p* < 0.05) and IgG1, and the IgG1/IgG2a was 1.78 (Figure 4).

### 2.5. Simvastatin-Enhanced Cellular Immune Responses Induced by rHis-SseB

**Figure 5:**
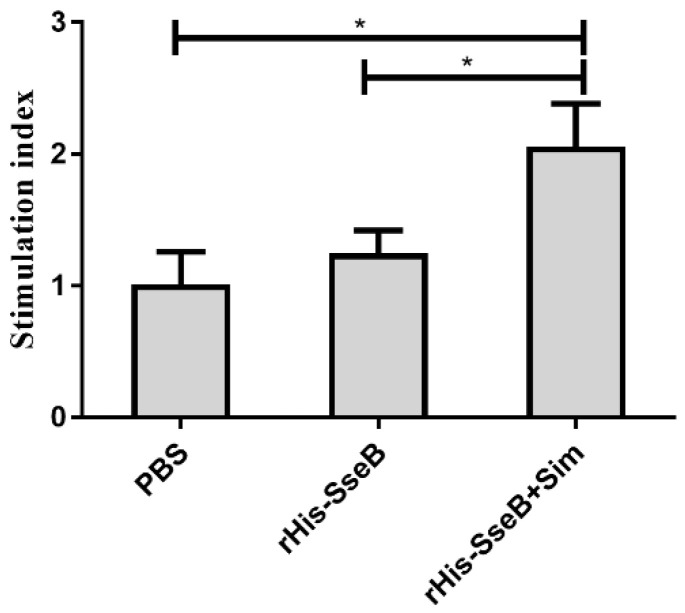
Stimulation index (SI) of the splenic lymphocytes proliferation assay. Splenic lymphocytes were isolated from vaccinated mice and stimulated with 10 μg/mL rGST-SseB for 72 h. Cell proliferation was determined by ELISA-BrdU. The SI was calculated using the following equation: SI = (OD_450_ − OD_690_ of the antigen-stimulated cells)/(OD_450_ − OD_690_ of the unstimulated cells). Data are presented as mean ± SEM, * *p* < 0.05.

Cellular immune responses were evaluated by measuring splenic lymphocyte proliferation 10 days after the second immunization. Splenic lymphocytes were isolated from vaccinated mice and stimulated with rGST-SseB for 72 h, and cell proliferation was measured using BrdU assays. Cell proliferation results show that the average stimulation index (SI) value of splenic lymphocytes in the rHis-SseB+Sim group was 2.06. The SI values of the splenic lymphocytes in the rHis-SseB+Sim group were significantly higher than those in the rHis-SseB alone and PBS groups (*p* < 0.05) (Figure 5).

### 2.6. Candidate Vaccine Protected Mice against Oral Challenge with Wild-type S. Enteritidis

**Figure 6:**
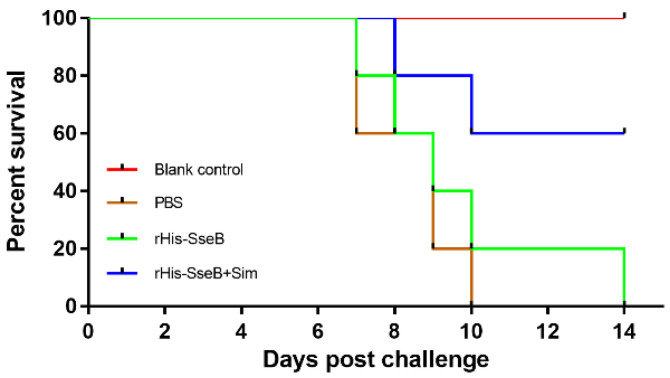
Protective efficacy of subunit vaccine against *S*. Enteritidis infection in mice. Mice were i.m. immunized either with 50 μg rHis-SseB (rHis-SseB group), 50 μg rHis-SseB mixed with 50 μg simvastatin (rHis-SseB+Sim group), or PBS on days 0 and 14. At two weeks after the second immunization (on day 28), the mice were challenged orally with 1 × 10^6^ CFU of virulent *Salmonella* Enteritidis strain C50041 in 100 μL of PBS. The number of mice surviving in each group was assessed in the following 2 weeks.

**Figure 7:**
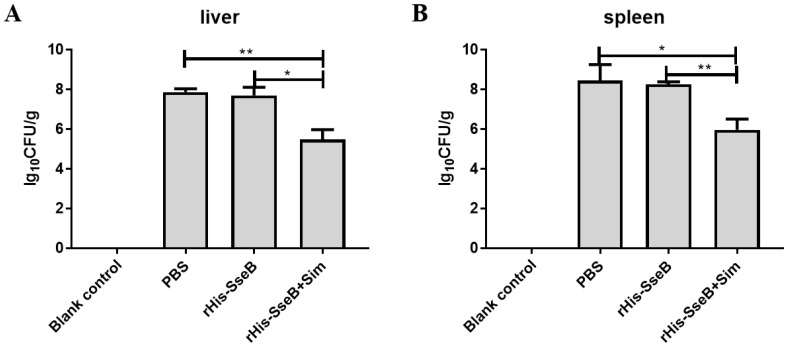
Bacterial colonization in mouse liver (**A**) and spleen (**B**) 4 days post *S*. Enteritidis challenge. The liver and spleen were aseptically collected from each group. Then, the samples were weighed and homogenized in 1 mL of PBS. The homogenates were serially diluted 10-fold and subsequently inoculated onto the LB agar plates at 37 °C for 12–16 h. The number of bacterial colonies was calculated as log_10_ CFU/g. Data are presented as mean ± SEM, * *p* < 0.05; ** *p* < 0.01.

To assess the protective efficacy of the candidate vaccine, mice were intramuscularly (i.m.) immunized twice and challenged orally with 1 × 10^6^ CFU of virulent *Salmonella* Enteritidis strain C50041. The survival rate of mice in the rHis-SseB+Sim group was 60%. However, all mice in the rHis-SseB and PBS groups died (Figure 6). Furthermore, compared with the rHis-SseB-immunized and PBS groups, delayed death of mice was observed in the groups co-immunized with rHis-SseB and simvastatin (Figure 6). We also measured the bacterial loads in the liver and spleen four days after the challenge. As shown in Figure 7A,B, compared with the rHis-SseB-immunized and PBS groups, the rHis-SseB+Sim group had reduced bacterial loads in both the liver and spleen.

## 3. Discussion

Salmonellosis is one of the most important zoonoses and foodborne illnesses, and is a major public health problem worldwide. Salmonellosis leads to heavy economic loss and is a threat to human health [25,26]. *S*. Enteritidis is one of the most common serotypes of salmonellosis in humans, despite the implementation of control and prevention measures [27]. Vaccination is an important and effective method to control and prevent salmonellosis. To date, a number of *S*. Enteritidis vaccines have proven effective in controlling and preventing *S*. Enteritidis infection, and they are used commercially worldwide [10]. However, most of these commercial vaccines are live-attenuated or killed. Ideally, the vaccine should first be safe [28]. Subunit vaccines are safe because they usually consist of defined antigens. In this study, we evaluated the immune response and protective efficiency of an *S*. Enteritidis candidate subunit vaccine based on the antigen SseB adjuvant with simvastatin.

Subunit vaccines usually require an appropriate adjuvant to enhance their ability to induce an immune response. The adjuvanticity of simvastatin has been confirmed in both mice and monkeys in a recent study [24]. Moreover, simvastatin has better adjuvanticity than adjuvants that are currently in clinical use or in active trials (alum, MF59, MPL, and polyethyleneimine) [24]. Simvastatin was used as an adjuvant to enhance the immune response induced by rHis-SseB. The rHis-SseB+Sim group elicited a robust antibody response and produced significantly higher IgG and IgG subtype titers, suggesting that simvastatin could be used as an adjuvant to enhance the immune response to rHis-SseB. Xia et al. also demonstrated that simvastatin could enhance the immunogenicity of target antigens (OVA, HPV16 E7, HBV surface antigen, and Influenza A/PR8 hemagglutinin A1) and induce a stronger IgG response, which is consistent with our results [24]. The IgG1/IgG2a subtype ratio reflects the immune response induced by a target antigen. A ratio of IgG1/IgG2a less than 0.5 indicates a Th1-biased response induced by the antigen. The ratio was between 0.5 and 2.0 shows that the response between Th1 and Th2a was balanced. A ratio of IgG1/IgG2a more than 2.0 indicates a Th2-biased response [29]. To determine the immune response induced by rHis-SseB, the rHis-SseB-specific IgG subtype was measured 14 days after the second immunization. In the rHis-SseB+Sim group, the SseB-specific IgG1 titers were significantly higher than the IgG2a titers (*p* < 0.05), and the ratio of IgG1/IgG2a was 4.75, suggesting that the immune response induced by rHis-SseB was Th2-biased.

To control the spread of *Salmonella*, apart from the humoral immune response, the cellular immune response is crucial to eradicate the intracellular *Salmonella* [30]. We evaluated the cellular immune responses by measuring splenic lymphocyte proliferation. Compared to the rHis-SseB and PBS groups, the significantly higher SI values in the rHis-SseB+Sim group indicates that simvastatin enhanced the rHis-SseB-specific cellular immune responses. These results suggest that simvastatin is an effective adjuvant to enhance humoral and cellular immune responses induced by rHis-SseB.

Protective efficacy is an important indicator in vaccine evaluation [31]. We evaluated the survival percentage of vaccinated mice after virulent *Salmonella* Enteritidis challenge. Co-immunization with rHis-SseB and simvastatin protected 60% of mice from *S*. Enteritidis challenge and delayed their death. This result is consistent with a previous study reporting that immunization with SseB provides mice with a modest degree of protection against *Salmonella* Typhimurium infection [32,33]. Decreased colonization of host tissues and organs by bacteria is another way to evaluate the protective ability of vaccines [34]. The number of *Salmonella* invading the liver and spleen in the group immunized with rHis-SseB and simvastatin significantly decreased compared to that in the PBS group at 4 days post *S*. Enteritidis challenge. These results suggest that rHis-SseB combined with simvastatin provides immune protection against *S*. Enteritidis infection and can be developed as a promising *S*. Enteritidis subunit vaccine candidate.

## 4. Materials and Methods

### 4.1. Mice and Ethics Statement

Six-week-old specific-pathogen-free female BALB/c mice were purchased from Beijing Vital River Laboratory Animal Technology Co., Ltd. (Beijing, China). The mice were kept in isolators and fed food and water that were all pathogen-free and exposed to a 12-h light/dark cycles; the temperature was maintained at 23 ± 1 °C. All animal experiments were approved by the Animal Welfare and Ethics Committees of Yangzhou University and complied with the Ethics Committee of Laboratory Animals and guidelines of the Institutional Administrative Committee (SYXK[Su]2017-0044).

### 4.2. Construction of Recombinant Expression Plasmid

Bacterial genomic DNA was extracted from *Salmonella* Enteritidis C50041 using the TIANamp Bacteria DNA Kit (Tiangen Biotech Co., Ltd., Beijing, China) according to the manufacturer’s instructions. To generate the recombinant expression plasmid pCold-*SseB*, the open reading frame (ORF) of the *SseB* gene (GenBank ID: ALV17802.1) was amplified by PCR with primers his-*SseB*-F and his-*SseB*-R (Table 1), using *Salmonella* Enteritidis genomic DNA as a template. Restriction enzyme sites *Kpn* I and *Eco*R I were introduced into primers his-*SseB*-F and his-*SseB*-R, respectively (underlined). The amplified *SseB* gene was inserted into a linearized pCold vector (digested with *Kpn* I and *Eco*R I) to create pCold-*SseB* based on homologous recombination technology using the ClonExpress II One Step Cloning Kit (Vazyme, Nanjing, China) according to the manufacturer’s instructions. To generate the recombinant expression plasmid pGEX-6p-1-*SseB*, the coding region of *SseB* was amplified with primers GST-*SseB*-F and GST-*SseB*-R, which introduced *Bam*H I and *Eco*R I sites (underlined parts), respectively (Table 1). The amplified *SseB* gene was inserted into a linearized pGEX-6p-1 vector (digested with *Bam*H I and *Eco*R I, Takara, Dalian, China) to create pGEX-6p-1-*SseB* using the ClonExpress II One Step Cloning Kit following the manufacturer’s protocol. The recombinant expression plasmids pCold-*SseB* and pGEX-6p-1-*SseB* were confirmed by restriction endonuclease digestion and DNA sequencing.

### 4.3. Expression and Purification of Recombinant Proteins rHis-SseB and rGST-SseB

The recombinant expression plasmids pCold-*SseB* and pGEX-6p-1-*SseB* were transformed into competent *E. coli* BL21 (DE3) cells. For the expression of rHis-SseB with a His tag at the end of its N-terminuses, *E. coli* BL21 (DE3) harboring pCold-SseB was induced by 0.5 mM isopropyl-β-d-1-thiogalactopyranoside (IPTG) at 15 °C for 24 h. For the expression of rGST-SseB with a GST tag at the end of its N-terminuses, *E. coli* BL21 (DE3) harboring pGEX-6p-1-*SseB* was induced by 0.5 mM IPTG at 30 °C for 5 h. The bacteria were harvested and resuspended in ice-cold PBS. The bacterial cells were lysed using ultrasonic tissue homogenizers. The bacterial lysates were evaluated using SDS-PAGE. The soluble recombinant proteins, rHis-SseB and rGST-SseB, were purified using the His•Bind^®^ Purification Kit (Novagen, San Diego, CA, USA) and GST Fusion Protein Purification Kit (GenScript, Nanjing, China).

### 4.4. Western Blotting

Recombinant proteins rHis-SseB and rGST-SseB were analyzed by western blotting using the monoclonal anti-His tag (Sigma, Saint Louis, MO, USA), GST mouse mAb (Cell Signaling Technology, Beverly, MA, USA), or antisera against *Salmonella* Enteritidis C50041 prepared by orally infecting mice with *Salmonella* Enteritidis C50041. Western blotting was performed as described previously [19]. Briefly, the contents of the recombinant proteins were separated by SDS-PAGE and electrotransferred to a nitrocellulose membrane. The membranes were probed with a monoclonal anti-His tag (1:3000), anti-GST tag mouse mAb (1:3000), or antisera against *Salmonella* Enteritidis C50041 (1: 1000) as the primary antibody and then incubated with horseradish peroxidase (HRP)-conjugated goat anti-mouse immunoglobulin G (IgG) antibody (1:5000 dilution; Sigma). Immunoreactivity was measured using Super Signal West Pico Chemiluminescent Substrate (Pierce, Chemical, Rockford, IL, USA) according to the manufacturer’s instructions.

### 4.5. Mouse Vaccination

Eighteen 6-week-old BALB/c mice were stochastically divided into three groups (*n* = 6). Mice were i.m. immunized with either 50 μg rHis-SseB (rHis-SseB group), 50 μg rHis-SseB mixed with 50 μg simvastatin (Sigma) (rHis-SseB+Sim group), or PBS. Mice were vaccinated twice, on days 0 and 14. Blood samples were collected on days 7, 14, 21, 28, and 35 via retro-orbital plexus puncture. The serum was obtained following centrifugation and stored at −70 °C.

### 4.6. Detection of Anti-SseB Antibodies by Indirect ELISA

SseB-specific IgG and IgG subtypes IgG1, IgG2a, and IgG2b were detected using indirect ELISA as described previously [35]. Briefly, 2 μg/mL rGST-SseB were regarded as coated antigen dissolved in 50 mM carbonate buffer in 96-well plates at 4 °C for 14 h. After washing and blocking, serial double dilutions (beginning at 1:100) of the serum samples were added to rGST-SseB covered 96-well plates for 2 h at 37 °C. After washing, HRP-conjugated goat anti-mouse IgG (1:10,000, Southern Biotech, Birmingham, AL, USA), IgG1 (1:3000, Southern Biotech), IgG2a (1:3000, Southern Biotech), or IgG2b (1:3000, Southern Biotech) were added for 1 h at 37 °C. After washing, 100 μL of 3,3′,5,5′-tetramethylbenzidine substrate was added to the 96-well plates for ELISA development and then stopped with 2 M H_2_SO_4_. Finally, the absorbance was measured at 450 nm.

### 4.7. Lymphocyte Proliferation Assay

Splenic lymphocyte proliferation was measured 10 days after the second immunization. Splenic lymphocytes were obtained from each mouse (*n* = 3) using density gradient centrifugation with Lymphoprep (specific gravity 1.077) (Sigma), as previously described [36]. Splenic lymphocyte suspensions (1 × 10^6^ cells/100 μL/well) were seeded into 96-well tissue culture plates and stimulated with 10 μg/mL rGST-SseB for 72 h. Cell proliferation was evaluated using an ELISA-BrdU kit (Roche, Basel, Switzerland), according to the manufacturer’s instructions. Cell proliferation was expressed as the stimulation index (SI) and calculated using the following equation: SI = (OD_450_ − OD_690_ of antigen-stimulated cells)/(OD_450_−OD_690_ of unstimulated cells).

### 4.8. Immune Protection Assessment

Thirty-two 6-week-old BALB/C mice were stochastically divided into four groups (*n* = 8). To assess the protective efficacy of the candidate vaccine, mice were i.m. immunized with 50 μg rHis-SseB (rHis-SseB group), 50 μg rHis-SseB mixed with 50 μg simvastatin (rHis-SseB+Sim group), or PBS on days 0 and 14. Two weeks after the second immunization (on day 28), the mice were challenged orally with 1 × 10^6^ CFU of virulent *Salmonella* Enteritidis strain C50041 in 100 μL of PBS. The PBS-immunized group without challenge was used as the blank control. The survival of the mice was monitored daily for 14 days. Four days after the challenge, the bacterial loads in the liver and spleen of three mice in each group were measured. The samples were aseptically collected, weighed, and homogenized in 1 mL of PBS. The homogenates were diluted 10-fold serially and subsequently inoculated onto the LB agar plates at 37 °C for 12–16 h. Bacterial colonies were calculated as log_10_ CFU/g.

### 4.9. Statistical Analysis

All experimental data were analyzed by unpaired Student’s *t*-test using Prism software (version 7.0; GraphPad Inc., San Diego, CA, USA). The values are expressed as the mean± SEM, and significant differences were assigned to *p* values < 0.05, and <0.01 denoted by * and **, respectively.

## 5. Conclusions

In conclusion, we developed an *S*. Enteritidis subunit vaccine candidate based on rHis-SseB and simvastatin. Simvastatin was used as an adjuvant to enhance SseB-specific humoral and cellular immune responses. Co-immunization with rHis-SseB and simvastatin provided immune protection against *S*. Enteritidis infection and decreased bacterial colonization in organs. These results indicate that rHis-SseB combined with simvastatin could be a subunit vaccine candidate for salmonellosis.

## Figures and Tables

**Table 1:** Primers used in this study.

Primer Name	Primer Sequences (5′→3′)	Restricted Site	Usage
his-*SseB*-F	AAGGTAGGCATATGGAGCTCGGTACCATGTCTTCAGGAAACATCTT	*Kpn* I	Construction of pCold-*SseB*
his-*SseB*-R	GACTGCAGGTCGACAAGCTTGAATTCTCATGAGTACGTTTTCTG	*Eco*R I	
GST- *SseB*-F	TTCTGTTCCAGGGGCCCCTGGGATCCATGTCTTCAGGAAACATCTT	*Bam*H I	Construction of pGEX-6p-1-*SseB*
GST- *SseB*-R	GGCCGCTCGAGTCGACCCGGGAATTCTCATGAGTACGTTTTCTGC	*Eco*R I	

Note: Restriction site underlined.

## Data Availability

The data supporting the conclusions are contained within the article.
